# Maternal fish and shellfish consumption and wheeze, eczema and food allergy at age two: a prospective cohort study in Brittany, France

**DOI:** 10.1186/1476-069X-12-102

**Published:** 2013-12-02

**Authors:** Fabienne Pelé, Emma Bajeux, Hélène Gendron, Christine Monfort, Florence Rouget, Luc Multigner, Jean-François Viel, Sylvaine Cordier

**Affiliations:** 1 Inserm (Institut National de la Santé et de la Recherche Médicale) UMR 1085, IRSET (Institut de Recherche Santé Environnement & Travail), Université de Rennes 1, Rennes, Cedex F-35042, France; 2 Faculté de Médecine, Université de Rennes 1, Rennes F-35043, France; 3 Centre Hospitalier Universitaire de Rennes (CHU), Service d’Epidémiologie et de Santé Publique, Rennes F-35033, France; 4 Département de Pédiatrie, Centre Hospitalier Universitaire de Rennes (CHU), Rennes, Rennes F-35033, France

**Keywords:** Fish intake, Shellfish intake, Pregnancy, Wheeze, Allergy, Children

## Abstract

**Background:**

Environmental exposures, including dietary contaminants, may influence the developing immune system. This study assesses the association between maternal pre-parturition consumption of seafood and wheeze, eczema, and food allergy in preschool children. Fish and shellfish were studied separately as they differ according to their levels of omega-3 polyunsaturated fatty acids (which have anti-allergic properties) and their levels of contaminants.

**Methods:**

The PELAGIE cohort included 3421 women recruited at the beginning of pregnancy. Maternal fish and shellfish intake was measured at inclusion by a food frequency questionnaire. Wheeze, eczema, and food allergy were evaluated by a questionnaire completed by the mother when the child was 2 years old (n = 1500). Examination of the associations between seafood intake and outcomes took major confounders into account. Complementary sensitivity analyses with multiple imputation enabled us to handle missing data, due mostly to attrition.

**Results:**

Moderate maternal pre-parturition fish intake (1 to 4 times a month) was, at borderline significance, associated with a lower risk of wheeze (adjusted OR = 0.69 (0.45-1.05)) before age 2, compared with low intake (< once/month). This result was not, however, consistent: after multiple imputation, the adjusted OR was 0.86 (0.63-1.17). Shellfish intake at least once a month was associated with a higher risk of food allergy before age 2 (adjusted OR = 1.62 (1.11-2.37)) compared to low or no intake (< once/month). Multiple imputation confirmed this association (adjusted OR = 1.52 (1.05-2.21)).

**Conclusions:**

This study suggests that maternal pre-parturition shellfish consumption may increase the risk of food allergy. Further large-scale epidemiologic studies are needed to corroborate these results, identify the contaminants or components of shellfish responsible for the effects observed, determine the persistence of the associations seen at age 2, and investigate potential associations with health effects observable at later ages, such as allergic asthma.

## Background

The developing immune system is known to be very sensitive to environmental chemicals [1,2] and the antenatal and early childhood period is an important window of vulnerability [3]. Prenatal exposure to environmental contaminants may increase IgE levels in cord blood [4,5] and in early childhood [6] and may be associated with respiratory or allergic symptoms in preschool children (wheezing or food allergy) [7,8]. Some of these contaminants are present in seafood, including polychlorinated biphenyls (PCBs), dioxins, other polychlorinated compounds, perfluorinated chemicals (PFCs), and metals [9,10]. On the other hand, seafood is a major source of long-chain polyunsaturated fatty acids (PUFAs), especially n-3 PUFAs, gestational exposure to which is well known to have beneficial effects on children’s subsequent health [11]. Moreover, there is growing evidence that n-3 PUFAs have anti-inflammatory properties and may modulate immune responses [12,13]. The positive effect of these PUFAs may therefore counterbalance in part the deleterious impact of contaminants. The levels of n-3 PUFAs (higher in fish than shellfish) and contaminants (often high in shellfish) however, differ between fish and shellfish [9,10]. Accordingly, these categories of seafood should be assessed separately.

The PELAGIE cohort of pregnant women living in Brittany (the western part of France surrounded by the sea), is particularly suitable for such an assessment, as shellfish consumption is sufficiently high to allow fish and shellfish effects to be disentangled.

The objective of this study was to estimate the association between fish and shellfish intake during pregnancy on wheeze, eczema and food allergy in early childhood.

## Methods

### Study population

The PELAGIE mother-child cohort study, fully described elsewhere [14,15] enrolled 3421 pregnant women before 19 weeks of gestation in three districts of Brittany (France), from 2002 through 2006. At inclusion, women received a questionnaire to complete at home about family, social, and demographic characteristics, as well as diet and lifestyle. They subsequently gave birth to 3323 liveborn singletons who were eligible for follow-up at two years of age. Around the children’s second birthday, 2996 questionnaires were sent to families, excluding those whose child (n = 6) or mother (n = 1) had died in the meantime and the 320 children of the 2002 cohort who were already older than 2.5 years on the date follow-up began. The questionnaires were intended to document the families’ socioeconomic and demographic characteristics and provide information about the health and lifestyle of the children and their parents. Of the 2996 eligible mothers, 770 could not be reached because of incorrect or no-longer-valid mailing addresses. Of the remaining 2226 mothers, 1505 (68%) mailed the completed questionnaire back to us. Maternal fish and shellfish consumption was available for 1500 of them. Those mothers provided informed written consent, and the INSERM ethics committee approved the study procedures.

### Assessment of fish and shellfish intake

At inclusion, women were asked to fill out a food frequency questionnaire that inquired about their usual consumption, before pregnancy, of 18 specific categories of food, originally selected because of their contribution to intake of polychlorinated dibenzo-dioxins/furans in the French population [16]. Seafood consumption was evaluated through 4 items: saltwater fish (including salmon), mollusks (oysters, mussels, etc.), large crustaceans (crabs, spider crabs, etc.), and small crustaceans (shrimp, etc.). For each of these 4 items, women reported their frequency of consumption on a five-point scale. Due to small numbers, fish intake was regrouped into 3 categories: “never or less than once a month”, “1 to 4 times a month” and “more than 4 times a month”. Shellfish consumption included mollusks and both large and small crustaceans in 2 categories: “never or less than once a month” and “at least once a month”.

### Assessment of wheeze, eczema, and food allergy

At the 2-year follow-up, the child’s principal caregiver, usually the mother (98%), completed a questionnaire aimed at evaluating the child’s health since birth. Three outcomes were considered: wheeze, eczema, and food allergy.

Wheeze was identified with 5 questions. Two were adapted from the International Study of Asthma and Allergies in Childhood (ISAAC) [17] questionnaire: (1) “Has your child ever had wheezing or whistling in the chest at any time in the past?” and (2) “Has your child ever had a medical diagnosis of asthma?” The three other questions sought to complete the wheeze assessment: (3) “Did your child have an asthma attack before the age of 1 year?” (4) “Has your child had an asthma attack since he or she reached the age of 1 year?” and (5) “Has your child ever had bronchiolitis or bronchitis?” Positive responses to at least one of the first 4 questions classified the child in the “certain wheeze” category. The “no wheeze” group included all children for whom all responses were negative. In France, wheeze before the age of two is often reported as bronchiolitis, while bronchitis does not involve wheeze symptoms. As the fifth question mixes these two diagnoses, it did not allow us to disentangle wheezing and nonwheezing children. A positive response to this question only therefore led us to place the child in the “probable wheeze” category.

Eczema was identified by a positive answer to both of the following questions (from the ISAAC questionnaire): “Has your child ever had an itchy skin rash, which was coming and going?” and “If yes, has this itchy rash affected any of the following places - the folds of the elbows, behind the knees, in front of the ankles, under the buttocks, or around the neck, ears, or eyes?” A positive answer to the single question “Has your child ever had a medical diagnosis of eczema?” also identified the presence of eczema.

The presence of a food allergy was identified by a positive answer to any one of the following 3 questions: “Has your child ever had a medical diagnosis of cow’s milk allergy?” or “Has your child ever had a medical diagnosis of any food allergy?” or “Has your child ever had an allergic reaction after eating food (swollen lips or face, gastrointestinal response)?”. For the last question, time to the allergic reaction and the type of food involved were verified.

### Statistical analyses

Polytomous logistic regression was used to estimate the association between maternal pre-parturition seafood consumption and wheeze (“certain wheeze” and “probable wheeze” compared to “no wheeze”, the reference category) before age two. Binomial logistic regression was used for eczema and food allergy. Three models per outcome were performed to test the specific effects of fish or shellfish intake, separately and together. To estimate the adjusted effect of seafood consumption, the following known or suspected risk factors were considered for inclusion in the models: mother’s age (continuous), maternal education (≤12 years, >12 years), prenatal exposure to tobacco (0, 1 to 5 cigarettes/day, ≥5 cigarettes/day), folic acid supplementation (yes, no), family history of asthma/allergy (yes, no), child’s sex, cesarean delivery (yes, no), preterm birth (<37 weeks of gestation; yes, no), small-for-gestational age (<10th percentile of the French birth weight distribution for gestational age and sex; yes, no), feeding method during the first 3 months of life (exclusive formula feeding, mixed, exclusive breastfeeding), number of siblings at birth (0, 1, ≥2), contact with farm animals (yes, no), attendance at group daycare (yes, no), postnatal exposure to tobacco (0, 1 to 20 cigarettes/day, ≥20 cigarettes/day), dampness and/or mold at home (yes, no), and child’s age at follow-up (continuous). A family history of asthma/allergy and the child’s sex were included in all models as well as other potential confounders that were associated with one of the two exposure variables and one of the three outcome variables with a p-value <0.2 in the univariate analysis. Interactions with a family history of asthma/allergy, sex, and infant feeding were tested. To handle missing data for the covariates, a missing modality was coded.

To take attrition at follow-up into account, we verified the stability of the results with sensitivity analyses that used multiple imputation [18]. This simulation technique allows to replace the missing values (due mostly to attrition: outcomes and covariates), under the missing at random assumption, by *m* > 1 simulated versions. Imputed values of a variable are estimated conditionally on other variables and a rich imputation model that preserves a large number of associations is desirable [19]. To this end, variables recorded at inclusion and at birth have been used to impute missing values due to attrition (prenatal fish and shellfish intake, mother’s age, maternal education, prenatal exposure to tobacco, folic acid supplementation, child's sex, cesarean section, preterm birth, small-for-gestational age, and number of siblings at birth). In this study, 10 data sets were generated, with observed values identical but various imputed values across the data sets. We ran multivariate regressions with the same covariates we defined in the complete-case analysis. The results were then combined to produce estimates with standard deviations that incorporated missing-data uncertainty. Results are reported as odds ratios (ORs) with their 95% confidence intervals. SAS software version 9.3 (SAS institute, Inc., Cary, NC) was used for data analysis.

## Results

Compared with the 1500 participants, nonrespondents (n = 1496) were younger at the birth of the PELAGIE child (p < 0.001), less educated (p < 0.001), and more likely to smoke (p < 0.001). Shellfish consumption did not differ between respondents and nonrespondents but the latter were less likely to eat fish (p = 0.001) (Table 1).

**Table 1:** Comparaison between respondents and non-respondents to the 2-year follow-up questionnaire

**Variables**		**Respondents**	**Non-respondents**	p-value*
		**n = 1500**	**n =1496**	
		**No (%)**	**No (%)**	
**Maternal factors**				
Fish consumption				
	<1 time a month	252 (16.8)	307 (20.7)	0.001
	1–4 times a month	815 (54.3)	823 (55.5)	
	≥2 times a week	433 (28.9)	352 (23.8)	
Shellfish consumption				
	<1 time a month	1004 (66.9)	982 (65.9)	0.57
	≥1 time a month	496 (33.1)	507 (34.1)	
Age (years)				
	<28	445 (29.7)	535 (35.8)	<0.001
	28 ≤ age < 32	542 (36.1)	524 (35.0)	
	≥32	513 (34.2)	437 (29.2)	
Education				
	≤12 years	497 (33.2)	659 (44.2)	<0.001
	>12 years	1000 (66.8)	832 (55.8)	
Tobacco smoke at the beginning of pregnancy				
	No	1126 (75.6)	992 (67.2)	<0.001
	Yes	364 (24.4)	484 (32.8)	
Folic acid supplementation				
	Yes	251 (17.0)	235 (16.1)	0.50
	No	1225 (83.0)	1226 (83.9)	
**Child/perinatal factors**				
Child’s sex				
	Male	772 (51.5)	747 (50.0)	0.40
	Female	727 (48.5)	748 (50.0)	
Cesarean section				
	Yes	255 (17.3)	258 (17.6)	0.84
	No	1218 (82.7)	1208 (82.4)	
Preterm birth (<37 weeks of gestation)				
	Yes	49 (3.3)	60 (4.0)	0.26
	No	1447 (96.7)	1422 (96.0)	
Small-for-gestational age (<10th percentile)				
	Yes	97 (6.5)	94 (6.3)	0.84
	No	1401 (93.5)	1400 (93.7)	
No. of siblings at birth				
	0	645 (43.2)	667 (44.6)	0.68
	1	562 (37.6)	548 (36.7)	
	≥2	287 (19.2)	276 (18.5)	

*Chi square test.

The mean age of participating mothers was 30.4 (SD 4.2) years, and 67% had completed high school. Over 25% reported smoking at the beginning of pregnancy. Thirty percent of the children had a family history of asthma and/or allergy. Most were born at term, by vaginal delivery, with a normal birth weight for gestational age. The mean age of the children at follow-up was 26.6 (SD 2.2) months (Table 2).

**Table 2:** Characteristics of the study population and frequency of wheeze, eczema and food allergy according to those characteristics (n = 1500)

**Variables**		**Total No. (%)**	**Probable wheeze No. (%)**	**Certain wheeze No. (%)**	p-value*	**Eczema No. (%)**	p-value*	**Food allergy No. (%)**	p-value*
	**Maternal factors**								
Age (years)									
	<28	445 (29.7)	196 (44.5)	93 (21.14)	0.59	150 (33.9)	0.28	31 (7.0)	0.12
	28 ≤ age < 32	542 (36.1)	236 (43.7)	114 (21.11)		176 (32.8)		50 (9.3)	
	≥32	513 (34.2)	224 (43.8)	93 (18.0)		149 (29.3)		55 (10.9)	
Education									
	≤12 years	497 (33.2)	225 (45.6)	107 (21.7)	0.17	157 (31.8)	0.96	39 (8.0)	0.27
	>12 years	1000 (66.8)	430 (43.2)	192 (19.3)		317 (32.0)		97 (9.7))	
No. of cigarettes/day at beginning of pregnancy									
	0	1111 (74.3)	490 (44.4)	213 (19.3)	0.56	348 (31.6)	0.69	102 (9.2)	0.93
	1 to 5	205 (13.7)	85 (41.6)	50 (24.5)		64 (31.7)		19 (9.4)	
	>5	179 (12.0)	77 (43.0)	36 (20.1)		62 (34.8)		15 (8.4)	
Folic acid supplementation									
	Yes	251 (17.0)	102 (40.6)	51 (20.3)	0.44	91 (36.4)	0.11	27 (10.8)	0.30
	No	1225 (83.0)	543 (44.6)	245 (20.1)		378 (31.2)		106 (8.7)	
	**Child/perinatal factors**								
Familial history of asthma/allergy									
	Yes	371 (29.4)	161 (43.6)	100 (27.1)	<0.001	133 (36.3)	0.04	52 (14.1)	<0.001
	No	890 (70.6)	372 (42.1)	159 (18.0)		269 (30.4)		69 (7.8)	
Child’s sex									
	Male	772 (51.5)	349 (45.6)	182 (23.8)	<0.001	276 (36.0)	<0.001	79 (10.3)	0.10
	Female	728 (48.5)	307 (42.3)	117 (16.1)		199 (27.6)		57 (7.9)	
Cesarean section									
	Yes	255 (17.3)	122 (48.0)	53 (20.9)	0.17	74 (29.5)	0.40	22 (8.7)	0.83
	No	1218 (82.7)	523 (43.2)	236 (19.5)		389 (32.2)		110 (9.1)	
Preterm birth (<37 weeks of gestation)									
	Yes	49 (3.3)	26 (53.1)	16 (32.6)	0.003	17 (34.7)	0.68	4 (8.3)	0.84
	No	1450 (96.7)	629 (43.6)	283 (19.6)		458 (31.9)		132 (9.2)	
Small-for-gestational age (<10th percentile)									
	Yes	97 (6.5)	46 (47.9)	22 (22.9)	0.35	40 (41.7)	0.03	8 (8.2)	0.75
	No	1401 (93.5)	608 (43.6)	277 (19.9)		435 (31.3)		128 (9.2)	
No. of siblings at birth									
	0	645 (43.2)	297 (46.5)	100 (15.6)	0.004	213 (33.3)	0.43	60 (9.4)	0.51
	1	562 (37.6)	235 (42.0)	137 (24.5)		178 (32.0)		55 (9.8)	
	≥2	287 (19.2)	121 (42.3)	62 (21.7)		83 (29.0)		21 (7.4)	
Feeding during the first 3 months									
	Formula	506 (34.5)	210 (41.7)	107 (21.3)	0.67	171 (34.1)	0.02	50 (10.0)	0.23
	Mixed	434 (29.6)	201 (46.6)	81 (18.8)		150 (34.8)		45 (10.4)	
	Breast	528 (35.9)	233 (44.4)	104 (19.8)		144 (27.5)		39 (7.5)	
Farm animal contact									
	Yes	463 (31.0)	201 (43.9)	87 (19.0)	0.77	155 (33.7)	0.29	41 (8.9)	0.88
	No	1028 (69.0)	448 (43.7)	210 (20.5)		315 (30.9)		93 (9.1)	
Collective day care attendance									
	Yes	154 (11.0)	72 (47.4)	38 (25.0)	0.05	59 (38.6)	0.07	20 (13.2)	0.07
	No	1240 (89.0)	536 (43.5)	239 (19.4)		385 (31.3)		108 (8.7)	
Mold and/or dampness at home									
	Yes	46 (3.2)	23 (51.1)	7 (15.6)	0.60	14 (30.4)	0.80	11 (23.9)	<0.001
	No	1396 (96.8)	613 (44.1)	280 (20.2)		445 (32.1)		122 (8.8)	
No. of cigarettes/day smoked at home									
	0	880 (61.2)	392 (44.9)	161 (18.5)	0.11	285 (32.7)	0.53	82 (9.4)	0.86
	1 to 20	467 (32.50)	193 (41.4)	102 (21.9)		138 (29.8)		43 (9.3)	
	> 20	90 (6.3)	38 (42.2)	26 (28.9)		27 (30.0)		10 (11.1)	
Child’s age at follow-up (months)									
	22 ≤ age ≤ 24	390 (26.0)	144 (37.0)	65 (16.7)	<0.001	119 (30.7)	0.03	37 (9.66)	0.48
	24 < age ≤ 26	272 (18.1)	138 (50.9)	48 (17.7)		68 (25.2)		27 (10.0)	
	26 < age ≤ 28	445 (29.7)	192 (43.4)	101 (22.8)		152 (34.5)		44 (9.9)	
	>28	392 (26.1)	182 (46.9)	84 (21.6)		135 (34.8)		28 (7.2)	

*Chi square test.

Overall, 252 (17%) women reported eating fish either never or less than once a month, 815 (54%) 1 to 4 times a month, and 433 (29%) more than 4 times a month; 496 (33%) reported eating shellfish once a month or more. Fish and shellfish consumption were positively correlated (p < 0.001). The median age at introduction of fish to the child was 8 months (IQR: 7–12) and was not associated with prenatal seafood consumption (p-value of 0.67 and 0.58 for prenatal fish and shellfish intake respectively).

Wheeze status was assessed in 1491 children: 299 (20%) had certainly had at least one wheezing episode and another 656 (44%) probably had at least one. Eczema status was known for 1487 children, 475 (32%) of whom were classified positive. Among them 319 (67%) had been medically diagnosed with eczema. Food allergy status was known for 1487 children, 136 (9%) were classified with food allergy: 37 had a medical diagnosis of cow’s milk allergy, 41 a medical diagnosis of a food allergy, and 22 of both, while 36 children had apparent allergic reactions to food but no doctor’s diagnosis. Several children had several diagnoses, and the most important correlation was observed between eczema and food allergy (p < 0.001). The associations with potential risk factors are presented in Table 2.

The change in the ORs between the crude and adjusted estimates did not exceed 7%. Table 3 presents the adjusted estimates. A borderline significant association was observed between moderate pre-parturition fish consumption before pregnancy and a lower risk of “certain wheeze” during the first two years of life (aOR = 0.69 (0.45–1.05)). Including both types of seafood consumption in the model did not change the results. Maternal seafood consumption was not associated with eczema. Shellfish intake once a month or more was associated with a higher risk of a food allergy (aOR = 1.62 (1.11–2.37)). The association decreased slightly when both types of seafood were included. No interactions were observed.

**Table 3:** Adjusted association measured between seafood consumption and wheeze, eczema and food allergy

		**Probable wheeze**	**Certain wheeze**	**Eczema**	**Food allergy**
		**OR (95% CI)**	**OR (95% CI)**	**OR (95% CI)**	**OR (95% CI)**
**Complete-case analysis**					
Fish consumption*		1398		1395	1395
	<1 time a month	1	1	1	1
	1–4 times a month	0.83 (0.58-1.17)	0.69 (0.45-1.05)§	1.06 (0.77-1.48)	1.27 (0.72-2.24)
	≥2 times a week	1.04 (0.70-1.55)	0.90 (0.56-1.44)	0.89 (0.61-1.29)	1.48 (0.80-2.76)
Shellfish consumption*					
	<1 time a month	1	1	1	1
	≥1 time a month	1.08 (0.83-1.40)	1.08 (0.78-1.49)	1.09 (0.85-1.40)	1.62 (1.11-2.37)‡
Fish consumption**					
	<1 time a month	1	1	1	1
	1–4 times a month	0.82 (0.57-1.17)	0.68 (0.45-1.04)§	1.04 (0.75-1.45)	1.16 (0.65-2.07)
	≥2 times a week	1.02 (0.68-1.54)	0.87 (0.54-1.42)	0.85 (0.58-1.25)	1.27 (0.67-2.39)
Shellfish consumption**					
	<1 time a month	1	1	1	1
	≥1 time a month	1.06 (0.81-1.39)	1.08 (0.77-1.51)	1.13 (0.87-1.45)	1.57 (1.06-2.31)‡
**Multiple imputation analysis**					
Fish consumption**		2981		2981	2981
	<1 time a month	1	1	1	1
	1–4 times a month	0.90 (0.69-1.17)	0.86 (0.63-1.17)	1.03 (0.76-1.39)	1.13 (0.70-1.83)
	≥2 times a week	1.07 (0.78-1.47)	1.11 (0.75-1.67)	0.92 (0.58-1.46)	1.37 (0.79-2.39)
Shellfish consumption**					
	<1 time a month	1	1	1	1
	≥1 time a month	1.02 (0.80-1.31)	1.02 (0.81-1.30)	1.01 (0.76-1.34)	1.52 (1.05-2.21)‡

*Adjusted for: mother’s age, maternal education, folic acid supplementation, familial history of asthma/allergy, child’s sex, small-for-gestational age, infant’s method of feeding, day care attendance, post-natal exposure to tobacco and, child’s age at follow-up.

**Both fish and shellfish consumption were included in the model with the adjustment variables above.

§ p-value < 0.10; ‡ p-value < 0.05.

After multiple imputation (n = 2981; that is, 2996 families eligible for follow-up minus those for whom seafood consumption was not known), the association between prenatal fish consumption and wheeze was no longer significant (aOR = 0.86 (063–1.17)). On the other hand, multiple imputation confirmed the results for eczema and food allergy observed in the complete-case analysis (Table 3).

## Discussion

In this large, population-based birth cohort study, moderate maternal pre-parturition fish consumption may be associated with a lower risk of wheeze before age two, and no significant association was observed with eczema and food allergy. Shellfish consumption at least once a month was associated with a higher risk of a food allergy.

Shellfish consumption was four times higher in the PELAGIE population than at the national level [14,20]. This allowed us to disentangle the effects of these different seafoods and makes this study, to the best of our knowledge, the first to examine the influence of maternal pre-parturition shellfish consumption on food allergy. Moreover, this study of this highly educated population provided high quality questionnaire data. Its prospective longitudinal nature enabled us to characterize exposure long before maternal reports of outcomes and to collect substantial data about factors known to be associated with wheeze, eczema, and food allergy at the age of two.

A limitation of the study is the attrition at follow-up. Since nonrespondents at follow-up both ate fish less often and shared characteristics of families with a higher prevalence of child respiratory or allergic problems [21], their absence is likely to induce bias in the estimation of the association between maternal fish consumption during pregnancy and wheeze. Sensitivity analyses with multiple imputation appear to confirm this hypothesis. On the other hand, there was no follow-up selection according to shellfish intake, and multiple imputation endorsed the association between maternal shellfish consumption and food allergy. Seafood consumption was evaluated before pregnancy, and women may have modified their habits at the beginning of pregnancy, especially for mollusk intake (because of the infectious risks). However, the biological half-life of both fatty acids and most of the contaminants in seafood is long, and reports of usual diet (i.e., before pregnancy) are likely to represent exposure during pregnancy. Measures of wheeze and eczema were adapted from the ISAAC questionnaires. Although this method has been validated for assessments of children from the age of 7 years [17], it is widely used in epidemiological studies of younger children [22-25]. Reports of outcomes were not confirmed by clinical examination or biological markers for atopy, these unsupervised methods might have induced non-differential misclassification. We did not take childhood intake of fish into account for two principal reasons. First, the age at introduction of fish was not associated with prenatal seafood consumption in our study and therefore should not confound the association between prenatal seafood consumption and outcomes. Second, although some previous studies have observed that later introduction of fish may be associated with an increased risk of eczema or sensitization to food allergens [26,27], others have used detailed prospective data to argue that these associations may in fact result from reverse causation: parental suspicions of allergic reactions were likely to influence the age at introduction of fish [28]. These two arguments justify our decision not to take childhood intake of fish into account in studying this association.

Some studies have examined the effect of maternal fish intake during pregnancy on either eczema [22,23,29-31] or asthma/wheezing [22-24,29,32] in children. Four of the five studies that focused on eczema showed a decreased risk with a high consumption of fish (>1/week) during pregnancy. The study [22] that did not observe any association was from the cohort with the lowest average fish consumption. As in that study, the percentage of high fish consumers is low in our population; leading to a lack of statistical power that may explain the absence of significant findings for this association. In our study we did not observe a consistent association between maternal fish consumption during pregnancy and childhood wheeze. Two previous studies have observed a decreased risk of asthma/wheezing [29,32] in children of women who consume high levels of fish. Of the five studies on this subject, those two had the populations of pregnant women with the highest average fish consumption and asthma/wheezing evaluation at the oldest age (6 and 7 years), when asthma is easier to diagnose. It is hypothesized that eating fish may reduce the risk of respiratory and allergic outcomes because it is a major source of long-chain n-3 PUFAs and micronutrients such as selenium [11,33] that may have anti-inflammatory properties [12,13,34]. On the other hand, fish is contaminated by marine pollutants, mainly PCBs, dioxins and other polychlorinated compounds, and methylmercury [9,10]. Previous study has reported no link between prenatal methylmercury exposure and asthma or atopic dermatitis at age 7 [6]. However, two of the three epidemiological studies [7,8,35] that evaluated effects of prenatal exposure to PCBs, dioxins, or other polychlorinated compounds at age 2 noted an increased risk of wheezes [8] or food allergy [7] at higher levels of exposure, after adjustment for major confounding factors. To our knowledge, two published studies have suggested a link between maternal fish consumption and increased risk of allergies [22,36]. One focused on food allergy [36]. In their population, with a level of fish consumption similar to that in ours, they assessed food allergen sensitization by skin prick tests and observed that higher consumption of fish during pregnancy was associated with a higher risk of such sensitization. Their results must, however, be interpreted with caution as they were obtained in a cross-sectional study with a retrospective assessment of prenatal diet. The second of these studies focused on wheezing and eczema and was based on a cohort design. The population of this study, with its very low fish consumption, may not benefit of simultaneous n-3 PUFAs intake [11,22].

This study (n = 2796) also observed an increased risk of wheeze and eczema in children of women who ate shellfish during pregnancy [22]. Our findings were not significant for these associations, perhaps because of the smaller sample size; they nonetheless suggest an increased risk of food allergy. French national surveys [9,10] have showed that on average, shellfish is less contaminated by PCBs, dioxins or other polychlorinated compounds, or methylmercury than fish is. Other contaminants have been detected in shellfish at higher concentrations than in fish, in particular, metals such as lead, cadmium, arsenic (including a small proportion of inorganic and methylated forms considered toxic [37]), and organic compounds such as PFCs [9,10,38]. French national surveys [10,38] have also shown non-compliance with the regulatory shellfish threshold for cadmium or high exposure levels for cadmium and arsenic. Both metals have demonstrated their potential developmental immunotoxicity [39-42]. These contaminations, which were associated in shellfish with low concentrations of n-3 PUFAs, may therefore explain those findings.

## Conclusions

Shellfish intake was associated with a higher risk of food allergy. In line with our previous work in the PELAGIE cohort showing that shellfish consumption is associated with decreased fecundability [43] and decreased fetal growth [14], we suggest yet another potential adverse effect. Further large-scale epidemiological studies are needed to corroborate these results, identify the contaminants or components of shellfish responsible for the effects observed, determine the persistence of the associations seen at age 2, and investigate potential associations with health effects observable at later ages such as allergic asthma.

## Abbreviations

aOR: Adjusted odds ratio; CI: Confidence interval; IQR: Interquartile range; ISAAC: International study of asthma and allergies in childhood; OR: Odds ratio; PCBs: Polychlorinated biphenyls; PFCs: Perfluorinated chemicals; PUFAs: Polyunsaturated fatty acids; SD: Standard deviation; SGA: Small-for-gestational age.

## Competing interests

The authors declare that they have no competing interests.

## Authors’ contributions

FP conducted the statistical analyses and drafted the initial manuscript. EB and HG took part in the statistical analyses. CM and FR were responsible for the field study including questionnaires and medical data. LM and JFV reviewed and revised the manuscript. SC designed the study and supervised the overall project. She critically reviewed the manuscript. All authors read and approved the final manuscript as submitted.
